# Supplementary material for: Activation of Steroidogenesis, Anti-Apoptotic Activity, and Proliferation in Porcine Granulosa Cells by RUNX1 Is Negatively Regulated by H3K27me3 Transcriptional Repression
Source: Genes (Basel). 2020 Apr 30;11(5):495. doi: 10.3390/genes11050495 (PMC7290568; doi:10.3390/genes11050495)
Supplement: Supplementary file 1 [file genes-11-00495-s001.zip › Figure S1 H3K27me3 target RUNX1 prediction.docx]

Figure S1 the bioinformatic prediction of the binding site between H3K27me3 and RUNX1 promoter. **(a)** Align between the swine *RUNX1* sequence and human *RUNX1* homologous sequence. **(b)** The predicted H3K27me3 in *RUNX1* was in the reserve strain of chr.13 198653051-198653684 bp.
